# The Diagnostic Challenges of Disseminated Nocardiosis in a Post-Renal Transplant Patient: A Case Report

**DOI:** 10.3390/reports8030111

**Published:** 2025-07-17

**Authors:** Yi Lin, Minqi Xu, Helen Genis, Nisha Andany, Lina Chen

**Affiliations:** 1 Faculty of Arts & Science, University of Toronto, Toronto, ON M5S 1A1, Canada; yilin.lin@mail.utoronto.ca; 2 Department of Pathology and Molecular Medicine, Queen’s University, Kingston, ON K7L 2V7, Canada; minqi.xu@kingstonhsc.ca; 3 Department of Medicine, University of Toronto, Toronto, ON M5S 1A1, Canada; helen.genis@mail.utoronto.ca (H.G.); nisha.andany@sunnybrook.ca (N.A.); 4 Division of Infectious Diseases, Sunnybrook Health Sciences Centre, Toronto, ON M4N 3M5, Canada; 5 Department of Laboratory Medicine & Pathobiology, University of Toronto, Toronto, ON M5S 1A1, Canada; 6 Division of Anatomic Pathology, Sunnybrook Health Sciences Centre, Toronto, ON M4N 3M5, Canada

**Keywords:** case report, disseminated nocardiosis, immunocompromised patients, nocardiosis, organ transplant

## Abstract

Disseminated nocardiosis is a rare, life-threatening infection, often misdiagnosed due to its resemblance to other conditions. We report a case of disseminated nocardiosis in a 62-year-old post-renal transplant patient presenting with pulmonary, hepatic, and pancreatic lesions. Despite multiple negative bacterial cultures, a histopathological examination of the liver revealed necrotizing granulomas with filamentous microorganisms, ultimately identified as *Nocardia*. This case highlights diagnostic challenges and the importance of integrating microbiological, pathological, and radiographical findings to manage and diagnose disseminated nocardiosis infections in immunocompromised individuals.

## 1. Introduction

Nocardiosis is a rare but serious opportunistic infection caused by *Nocardia* species, a genus of aerobic, filamentous, Gram-positive bacteria [1,2]. Nocardiosis can involve multiple organs and poses a particular risk to immunocompromised individuals, such as organ transplant recipients or patients receiving immunosuppressive therapy [2]. Disseminated nocardiosis is a severe and widespread infection involving two or more organ systems; as such, clinical manifestations of nocardiosis are highly variable and depend on the site of infection [3,4]. Likewise, the diagnosis of nocardiosis is often delayed due to its nonspecific presentation and the resemblance of *Nocardia* to other microorganisms, such as *Actinomyces*, *Candidatus Savagella*, or fungi, in terms of morphology or staining patterns [5,6,7]. This report presents a challenging case of disseminated nocardiosis in a post-renal transplant patient, highlighting the importance of integrating a multidisciplinary approach that incorporates microbiological, pathological, and radiographical approaches to achieve accurate and timely diagnosis.

## 2. Case Presentation

A 62-year-old man presented to the hospital with a two-month history of malaise, fever, night sweats, anorexia, jaundice, and weight loss. His medical history was notable for chronic kidney disease, diabetes mellitus, hypothyroidism, dyslipidemia, gout, and hypertension. Six months prior to presentation, he had undergone renal transplantation and was on an immunosuppressive regimen consisting of tacrolimus and prednisone. He was originally from Bangladesh and underwent his renal transplantation in India; he had no known history of tuberculosis (TB) or exposure to TB and did not undergo screening for latent TB prior to transplantation.

Initial laboratory investigations revealed a normal total white blood cell (WBC) count with normal neutrophils but marked lymphopenia (0.06 × 10^9^/L, normal: 1.00–4.00 × 10^9^/L) and monocytopenia (0.09 × 10^9^/L, normal: 0.30–0.80 × 10^9^/L), consistent with significant immunosuppression. His liver profile showed normal alanine aminotransferase (ALT) but markedly elevated alkaline phosphatase (ALP) at 1046 U/L (normal: 40–120 U/L) and a total bilirubin level of 61 µmol/L (normal: <20 µmol/L), suggesting biliary obstruction and cholangitis.

In the hospital, radiological imaging revealed multiple lesions in the lungs, liver, and pancreas, raising concerns for infectious etiology. The pancreatic and liver lesions also resulted in biliary obstruction and cholangitis. The leading diagnostic consideration at that time was disseminated tuberculosis, with other bacterial, mycobacterial, and fungal etiologies also being included in the differential diagnoses. Multiple blood, stool, and urine cultures for bacteria, fungi, and mycobacteria came back negative.

Non-infectious etiologies such as neoplasm were also considered. He underwent multiple diagnostic procedures including endoscopic retrograde cholangiopancreatographies (ERCPs) and the fine-needle aspiration of pancreatic lesions with samples submitted for cytology and culture, as well as lung biopsy with samples submitted for culture and pathology. These tests were all non-diagnostic, with the lung biopsy showing only acute and chronic inflammation, with no evidence of granulomatous inflammation and no evidence of malignancy. As such, a targeted liver lesion biopsy was subsequently performed, and this was again sent for pathology as well as bacterial, fungal, and mycobacterial culture. These microbiological studies all came back negative.

**Figure 1:**
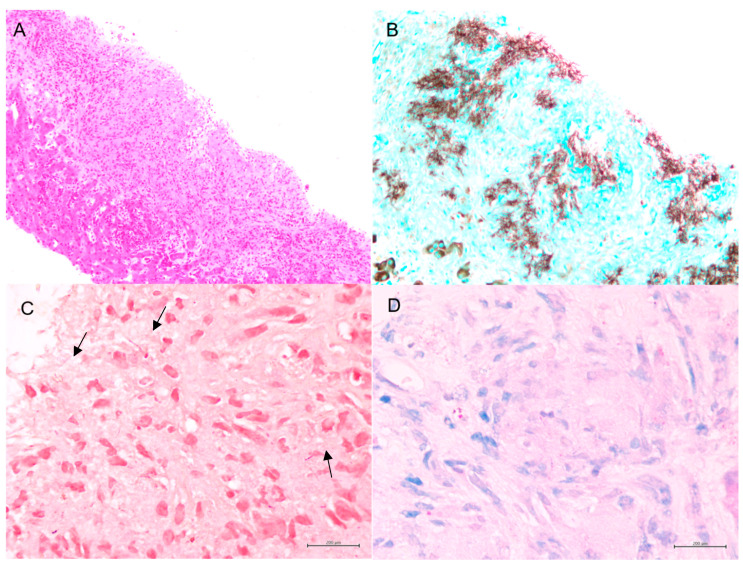
(**A**). Necrotizing granuloma in liver biopsy (HE stain, 50×). (**B**). Filamentous microorganisms with beaded morphology present in the necrotizing granuloma (GMS stain, 100×). (**C**). The microorganisms (some indicated by arrows) are Gram-positive (Gram stain, 400×). (**D**). The microorganisms show weak FITE stain (Fite stain, 400×).

**Figure 2:**
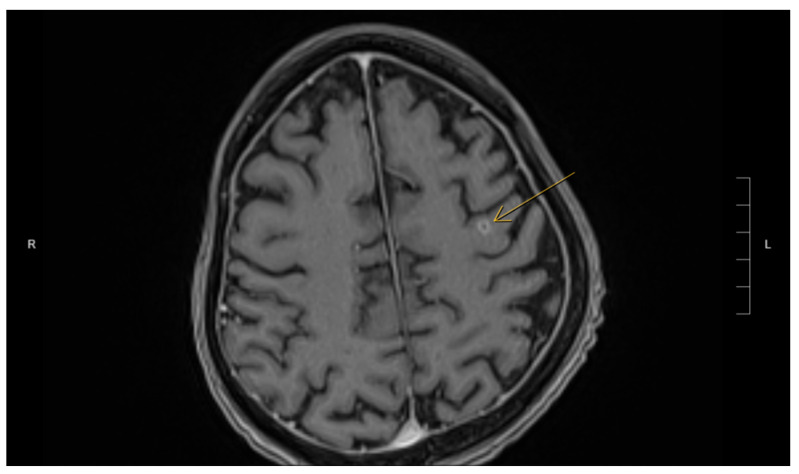
MRI of the brain demonstrating a 5 mm ring-enhancing lesion (indicated by arrow) in the left precentral gyrus.

**Figure 3:**
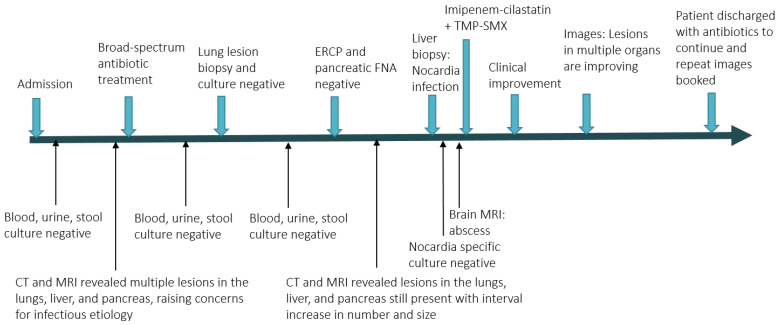
Timeline of the patient’s 3-month hospitalization with selected events.

Fortunately, the histopathological examination of the targeted liver biopsy revealed necrotizing granulomas containing filamentous microorganisms. The histomorphology findings and special stain patterns raised the possibility of *Nocardia* infection; however, there was no reliable way to identify the specific species. The beaded morphology seen on special stains, along with the weak FITE stain signal, were consistent with features typically associated with *Nocardia* (see Figure 1). At the same time, *Actinomyces* was considered less likely due to the patient’s poor clinical response to initial antibiotic treatment with amoxicillin–clavulanate, vancomycin, and piperacillin–tazobactam, which were provided for cholangitis and possible pyogenic liver abscess. No organisms were detected on Ziehl–Neelsen staining, but the possibility of superimposed mycobacterial infection, such as TB, could not be entirely excluded due to the limited sensitivity of the Ziehl–Neelsen stain. As a result, the sample was sent for polymerase chain reaction (PCR) testing for tuberculosis, which was negative. Brain imaging was suggested to rule out a central nervous system (CNS) abscess, as CNS involvement is more common in immunocompromised patients with *Nocardia* infection [8]. This demonstrated a 5 mm rim-enhancing lesion in the left pre-frontal gyrus (see Figure 2). The patient was presumptively diagnosed with disseminated nocardiosis. He was initiated on antimicrobial therapy with a combination of imipenem–cilastatin and trimethoprim–sulfamethoxazole (TMP-SMX). He completed 3 months of combination therapy and thereafter was transitioned to monotherapy with TMP-SMX, which he took for an additional 5 months (total 8 months of therapy). This was discontinued at 8 months due to the development of drug-induced liver injury. While receiving this antimicrobial regimen, he demonstrated clinical improvement and radiological resolution of his brain, lung, liver, and pancreatic lesions, clinically confirming the diagnosis. He has been followed in the outpatient Infectious Diseases clinic, and one year after the discontinuation of his antimicrobials, there have been no clinical nor radiographic concerns for relapse. Figure 3 shows the timeline of the patient’s 3-month hospitalization with some selected important events.

## 3. Discussion

This case highlights the diagnostic challenges associated with disseminated nocardiosis, a rare and potentially life-threatening infection that can affect both immunocompetent and immunocompromised hosts, as well as the utility of histopathologic examination in addition to microbiological testing for patients with undifferentiated infectious diseases syndromes. The clinical manifestations of nocardiosis are highly heterogeneous and nonspecific, and the infection can affect various organs including the lungs, brain, and skin, resulting in a diverse presentation that can be difficult to differentiate from other infections [9]. Filamentous microorganisms such as *Nocardia* can resemble other pathogens, such as *Candidatus savagella*, complicating diagnosis due to their shared filamentous or branching morphology [6,10]. This finding is consistent with numerous previous studies, which highlight the diagnostic difficulties posed by the morphological similarities between *Nocardia* and other filamentous pathogens [4]. *Actinomyces* and *Nocardia* also share similarities in that both can cause acute inflammation or pyogranulomatous reactions, characterized by a combination of acute inflammation and granuloma formation [10,11]. This common pathological feature further complicates their differentiation. These findings also align with previous studies that emphasize the overlapping inflammatory responses in infections caused by *Nocardia* and *Actinomyces*, making it challenging to distinguish between the two [4]. Additionally, *Nocardia*, like many fungi or *Actinomyces*, is Gram-positive and may produce similar staining results, such as positive staining with Grocott–Gomori methenamine silver (GMS) [11]. This further complicates the diagnostic process, underscoring the importance of combining histopathological evaluation with microbiological culture and molecular testing. As such, recognizing morphology through special stains, along with a thorough differential diagnosis, is essential for accurate identification. Clinicians must also be aware of the limitations of these techniques, as false-negative results can occur, particularly when relying on a single diagnostic method and whenever possible, should endeavor to pair histopathological analysis with microbiologic investigations.

Detection or growth of organisms through microbiologic testing is the gold standard for diagnosing bacterial infection. However, some microorganisms are fastidious and do not grow well or at all with standard bacteriological methods, which can result in falsely negative tests. It is important that the collection of samples and performance of microbiologic testing be coordinated in conjunction with the microbiology lab, to ensure that proper media and techniques are utilized. Molecular techniques, such as polymerase chain reaction, can also be useful in certain circumstances. In this case, after the liver histology diagnosis, the microbiology lab was asked to culture stool, blood, urine, and liver samples to isolate Nocardia; however, the cultures remained negative, with no growth even after five days and/or two weeks. The diagnosis was therefore made only based on histopathology, with the recognition of the bacteria’s histomorphological features proving key to establishing the diagnosis. Pathologists must be aware of the filamentous bacteria and their mimics. Filamentous bacteria can mimic fungi or mycobacteria both in morphology and special stains (GMS, ZN), and some bacteria can develop abnormal morphology (filamentation) after the administration of subinhibitory concentrations of antibacterial agents [12]. Table 1 summarizes the features of filamentous bacteria and fungal hyphae that are reported in the literature [13,14]. Table 2 lists the features of *Actinomyces* and *Nocardia* that are reported in the literature [2,12].

The management of disseminated and severe nocardiosis typically includes an initial phase of 2–3 active antimicrobial agents in combination, followed by consolidation therapy with a single agent. Antimicrobials that may be active against *Nocardia* species include amikacin, trimethoprim–sulfamethoxazole, linezolid, etc. TMP-SMX is generally a component of the initial treatment regimen and is often the agent of choice for consolidation monotherapy due to its reliable activity against most species of *Nocardia*. TMP-SMX is particularly effective in managing cerebral nocardiosis due to its excellent penetration into the central nervous system (CNS). However, treatment regimens may be chosen and adjusted based on the site of infection, the specific *Nocardia* species, antimicrobial susceptibility testing, and the patient’s response [11,13]. The duration of therapy is typically prolonged, often between 6 and 12 months. In this case, following the histological confirmation of *Nocardia* infection, the treatment was empirically adjusted to the combination of imipenem–cilastatin and TMP-SMX, as neither culture data nor antimicrobial susceptibility testing was available. This change resulted in rapid clinical improvement, underscoring the importance of a timely, appropriate antibiotic regimen and early diagnosis consistent with previous studies. However, past studies note that delays in diagnosis can worsen patient outcomes, a challenge in this case [4]. Most cases are not identified in the early stages due to nonspecific symptoms, and it can take anywhere from 42 days to several months for a definitive diagnosis. This delay can result in a considerable physical and emotional burden for patients, particularly when the infection involves multiple organ systems [3,5]. For instance, CNS involvement significantly affects prognosis, as mortality rates are markedly higher in patients with CNS lesions compared to those without [3,8]. This aligns with previous findings that suggest that CNS involvement in nocardiosis leads to poorer patient outcomes. Furthermore, studies indicate that the mortality rate is 10-fold higher in patients with solid organ transplantation due to nocardiosis compared to those without, highlighting the critical need for early diagnosis and intervention [10,11].

Overall, our case underscores several key clinical considerations. First, it emphasizes the importance of maintaining a high index of suspicion for nocardiosis, particularly in immunocompromised patients with multisystem involvement. This recommendation mirrors the conclusions of numerous studies that advocate for heightened awareness in immunocompromised populations. Second, it is crucial to recognize and differentiate *Nocardia* from other pathogens, such as mycobacteria and fungi. Finally, using a multidisciplinary approach that combines histopathological, radiological, and molecular diagnostic tools is vital for accurate identification to overcome the limitations of individual techniques.

## 4. Conclusions

In conclusion, early diagnosis and treatment can dramatically improve prognosis by mitigating complications associated with delayed diagnosis. Given the challenges in diagnosing this infection and the complexities of treating immunocompromised patients, a multidisciplinary approach, incorporating clinical, radiological, microbiological, and pathological tools, is essential for effective care. Ongoing research to refine diagnostic methods, treatment regimens, and strategies for managing immunosuppressive patients is needed to enhance outcomes for those facing this infection. These ongoing advancements are crucial for addressing the evolving challenges posed by nocardiosis.

## Figures and Tables

**Table 1:** The comparison between filamentous bacteria and fungal hyphae [13,14].

Characteristics	Filamentous Bacteria	Fungal Hyphae
Gram Stain Reaction	Gram-negative or -positive	Gram-positive
Special Staining Characteristics	GMS-positive	GMS-positive
Morphology (Shape and Size)	Delicate narrow filaments (up to 1 um)	Hyphae usually at least 2–3 um thick
Structure	Filaments may have right-angle branching	Various degrees of branching
Ecological Relevance	Tangled network of filamentous microorganisms	Scattered overlapping microorganisms

**Table 2:** The comparison between *Actinomyces* and *Nocardia* [2,12].

Characteristics	Actinomyces	Nocardia
Oxygen Requirement	Anaerobic	Anaerobic
Gram Stain Reaction	Gram-positive	Gram-positive
Special Staining Properties	GMS-positive	GMS-positive
Histopathology	Acute inflammation/pyogranulomatous	Acute inflammation/pyogranulomatous
Acid-fast Staining	Not acid-fast	Partially acid-fast
Clinical Presentation	Oral cervicofacial, uterus, disseminated	Lung, CNS, skin, hematogenous spread

## Data Availability

The data presented in this study are available on request from the corresponding author due to patient privacy.
